# A Deep-Learning Algorithm (ECG12Net) for Detecting Hypokalemia and Hyperkalemia by Electrocardiography: Algorithm Development

**DOI:** 10.2196/15931

**Published:** 2020-03-05

**Authors:** Chin-Sheng Lin, Chin Lin, Wen-Hui Fang, Chia-Jung Hsu, Sy-Jou Chen, Kuo-Hua Huang, Wei-Shiang Lin, Chien-Sung Tsai, Chih-Chun Kuo, Tom Chau, Stephen JH Yang, Shih-Hua Lin

**Affiliations:** 1 Division of Cardiology, Department of Medicine Tri-Service General Hospital National Defense Medical Center Taipei Taiwan; 2 Graduate Institute of Life Sciences National Defense Medical Center Taipei Taiwan; 3 School of Public Health National Defense Medical Center Taipei Taiwan; 4 Department of Research and Development National Defense Medical Center Taipei Taiwan; 5 Department of Family and Community Medicine Tri-Service General Hospital National Defense Medical Center Taipei Taiwan; 6 Planning and Management Office Tri-Service General Hospital National Defense Medical Center Taipei Taiwan; 7 Department of Emergency Medicine Tri-Service General Hospital National Defense Medical Center Taipei Taiwan; 8 Graduate Institute of Injury Prevention and Control College of Public Health and Nutrition Taipei Medical University Taipei Taiwan; 9 Division of Cardiovascular Surgery, Department of Surgery Tri-Service General Hospital National Defense Medical Center Taipei Taiwan; 10 Department of Electrical Engineering National Taiwan University Taipei Taiwan; 11 Department of Medicine Providence St Vincent Medical Center Portland, OR United States; 12 Department of Computer Science and Information Engineering National Central University Taoyuan Taiwan; 13 Division of Nephrology, Department of Medicine Tri-Service General Hospital National Defense Medical Center Taipei Taiwan

**Keywords:** artificial intelligence, sudden cardiac death, electrocardiogram, machine learning, potassium homeostasis

## Abstract

**Background:**

The detection of dyskalemias—hypokalemia and hyperkalemia—currently depends on laboratory tests. Since cardiac tissue is very sensitive to dyskalemia, electrocardiography (ECG) may be able to uncover clinically important dyskalemias before laboratory results.

**Objective:**

Our study aimed to develop a deep-learning model, ECG12Net, to detect dyskalemias based on ECG presentations and to evaluate the logic and performance of this model.

**Methods:**

Spanning from May 2011 to December 2016, 66,321 ECG records with corresponding serum potassium (K^+^) concentrations were obtained from 40,180 patients admitted to the emergency department. ECG12Net is an 82-layer convolutional neural network that estimates serum K^+^ concentration. Six clinicians—three emergency physicians and three cardiologists—participated in human-machine competition. Sensitivity, specificity, and balance accuracy were used to evaluate the performance of ECG12Net with that of these physicians.

**Results:**

In a human-machine competition including 300 ECGs of different serum K+ concentrations, the area under the curve for detecting hypokalemia and hyperkalemia with ECG12Net was 0.926 and 0.958, respectively, which was significantly better than that of our best clinicians. Moreover, in detecting hypokalemia and hyperkalemia, the sensitivities were 96.7% and 83.3%, respectively, and the specificities were 93.3% and 97.8%, respectively. In a test set including 13,222 ECGs, ECG12Net had a similar performance in terms of sensitivity for severe hypokalemia (95.6%) and severe hyperkalemia (84.5%), with a mean absolute error of 0.531. The specificities for detecting hypokalemia and hyperkalemia were 81.6% and 96.0%, respectively.

**Conclusions:**

A deep-learning model based on a 12-lead ECG may help physicians promptly recognize severe dyskalemias and thereby potentially reduce cardiac events.

## Introduction

Dyskalemias—hyperkalemia and hypokalemia—are common causes of sudden cardiac death in clinical practice [1]. Prompt recognition and rapid correction of these potassium (K^+^) derangements are needed to prevent catastrophic outcomes [2]. Currently, the detection of dyskalemia relies on laboratory tests. Point-of-care blood testing provides rapid analysis of electrolyte levels, however, its accuracy and precision may not be as reliable as that from a clinical central laboratory; this is mainly due to dilution, which would underestimate plasma K^+^ concentration, and the inability to discern hemolysis from pseudohyperkalemia [3,4]. Electrocardiography (ECG) is universally needed in patients with emergent cardiac or noncardiac conditions, which may exhibit the typical changes seen in dyskalemia since cardiac tissue is very sensitive to this disease. The main ECG changes associated with hypokalemia include a decreased T wave amplitude, ST-segment depression, T wave inversion, a prolonged PR interval, and an increased corrected QT interval (QTc) [5]. The typical ECG findings for hyperkalemia progress from tall *peaked* T waves and a shortened QT interval to a lengthened PR interval and a loss of the P wave, followed by a widening QRS complex and ultimately a *sine wave* morphology [5,6]. Although these morphologic changes are well known in dyskalemias, even experienced clinicians frequently do not notice all of these subtle details [7].

Previous researchers have developed ECG quantification algorithms to predict serum K^+^ concentration based on T wave morphology, mainly using the slope and width of T waves. Hyperkalemia is associated with tall, narrow, and symmetrical T waves, whereas hypokalemia is associated with flat T waves [8-12]. The algorithms were mostly derived from continuous patient monitoring, such as during hemodialysis, with homogeneous ECG morphologies from a limited set of patients [8-12]. Recently, applying the processing of T wave morphologies manually has been used to improve the diagnosis of hyperkalemia [13]. Nevertheless, using T wave changes alone to detect dyskalemias is less sensitive and specific than a comprehensive ECG interpretation [14].

With the revolution in artificial intelligence (AI), several advanced deep-learning models, such as Oxford’s VGGNet [15], Inception Net [16], ResNet [17], and DenseNet [18], have been developed, providing an unprecedented opportunity to improve health care; this was initiated by AlexNet’s victory in the ImageNet Large Scale Visual Recognition Challenge in 2012 [19]. Existing deep-learning models have been shown to achieve human-level performance and be effective in medical applications when large annotated datasets are available [17,20-22]. This potential to improve diagnosis and patient care prompted us to develop a deep-learning model to assist emergency physicians in recognizing ECG changes associated with dyskalemias.

Our study aimed to train a deep-learning model, ECG12Net, to predict serum K^+^ concentration by ECG. The deep-learning model was an 82-layer convolutional neural network that underwent a series of training processes to optimize model performance. The AI system, which will learn from more than 50,000 electrocardiograms to identify critical morphologic changes, will help to reduce medical errors in emergency departments (EDs) resulting from intense time pressure and harried ED staff during busy periods in ED environments [23]. Facilitated by the system’s powerful computing ability, the performance of the trained model was compared with that of emergency physicians and cardiologists. Finally, we visualized ECG12Net’s calculation process to understand why and how it works.

## Methods

### Data Source

The data were obtained from Tri-Service General Hospital, Taiwan, and research approval was given by the Institutional Review Board (IRB) (IRB No. 1-107-05-047). From May 11, 2011, to December 31, 2016, 40,180 emergency patients were enrolled who had 66,321 ECG records within 1 hour before or after serum K^+^ concentration for reference. Serum K^+^ concentrations were measured in the laboratory using indirect ion-selective electrode methods that had been accredited by the International Organization for Standardization (ISO) standard ISO-15189 and the College of American Pathologists’ Laboratory Accreditation Program. All hemolyzed samples were excluded. Potential confounders, such as patients with chest pain or thyroid disorders, were not excluded from the study. We divided the dataset into training (~70%), validation (~10%), and test (~20%) sets by date. Emergency patients presenting before April 30, 2016, were included in the training set; those presenting between May 1 and July 20, 2016, were in the validation set; and those presenting after July 21, 2016, were in the test set to assess model performance. All records included in the training set were excluded from the validation and test sets; thus, there was no overlap among the three datasets. The ECG recordings were collected using a Philips 12-Lead ECG machine (PH080A). The ECG signal was recorded in a digital format. The sampling frequency was 500 Hz with 2.5 seconds recorded in each lead. The estimated K^+^ concentrations ranged from 1.5 mEq/L to 7.5 mEq/L. Predicted K^+^ concentrations less than 1.5 mEq/L or greater than 7.5 mEq/L were indicated accordingly without further detail (ie, as either <1.5 mEq/L or >7.5 mEq/L). Patient characteristics and laboratory results were collected using an electronic health record system. The estimated glomerular filtration rate was calculated using the Chronic Kidney Disease Epidemiology Collaboration formula [24]. Eight basic ECG morphology parameters (EMPs) were calculated by the Philips 12-Lead ECG machine: heart rate, PR interval, QRS duration, QT interval, QTc, P wave axis, RS wave axis, and T wave axis.

### The Implementation of ECG12Net

**Figure 1:**
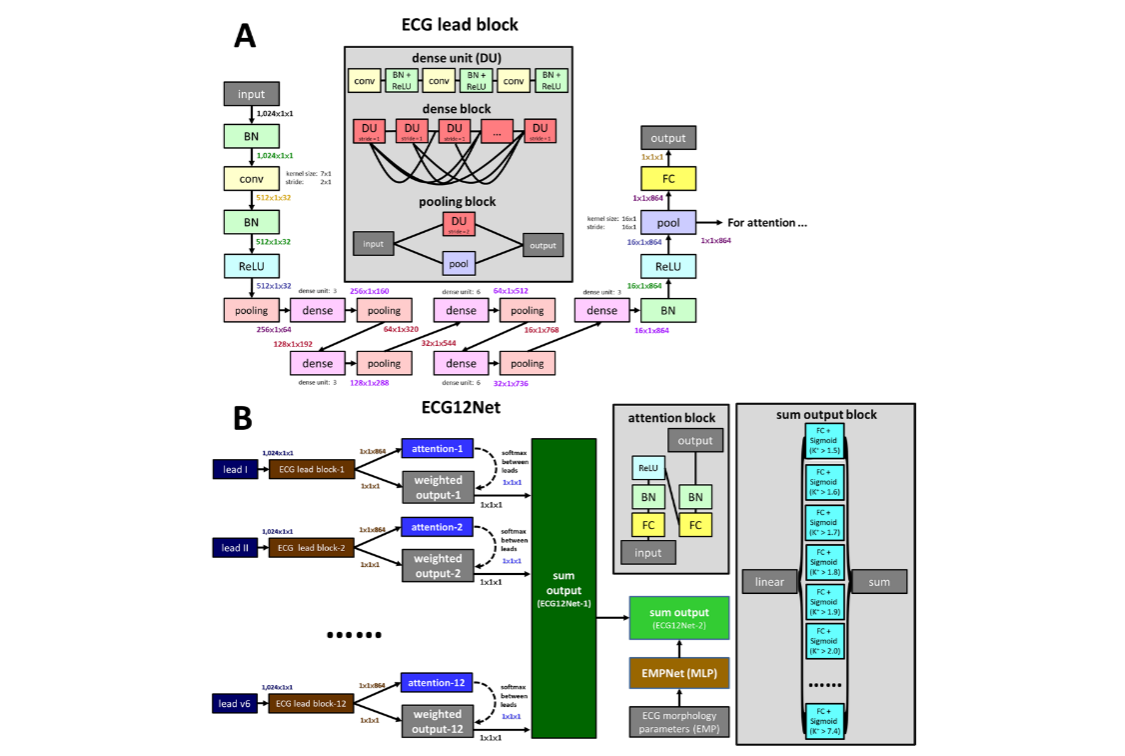
Architecture of ECG12Net. A. Electrocardiography (ECG) lead block with 80 trainable layers. B. ECG12Net integrates all the information from the ECG leads to make an overall prediction. The bolded and colored words indicate the output dimensions of the layers, and the words in black are the required parameters for the layers. conv: convolution; BN: batch normalization; ReLU: rectified linear unit; FC: fully connected; MLP: multilayer perceptron.

We developed a 12-channel sequence-to-sequence model, which is modified from DenseNet [18]. The details are shown in Multimedia Appendix 1. The architecture of ECG12Net is shown in Figure 1. We designed an ECG lead block with 80 trainable layers whose architecture is shown in Figure 1 A. This ECG lead block was used to extract 864 features from each ECG lead, making a basic output prediction based on each lead. Figure 1 B shows how ECG12Net integrates all the information from the ECG leads to make an overall prediction. ECG12Net is composed of 12 of these ECG lead blocks corresponding to each lead sequence. We designed an attention mechanism based on a hierarchical attention network to concatenate these blocks, increasing the interpretive power of ECG12Net [25]. ECG12Net-1, which uses only ECG wave information, contains 82 trainable layers. To improve prediction performance, we added an EMPNet, which is a multilayer perceptron with two hidden layers containing eight EMPs, to ECG12Net-1 to create ECG12Net-2.

### Human-Machine Competition

We evaluated the performance of practicing physicians using a subtest set. We divided the data into five categories based on the serum K^+^ concentration: (1) K^+^ ≤2.5 mEq/L, (2) 2.5< K^+^ ≤3.5 mEq/L, (3) 3.5< K^+^ <5.5 mEq/L, (4) 5.5≤ K^+^ <6.5 mEq/L, and (5) K^+^ ≥6.5 mEq/L. Stratified sampling was used to create the subtest set due to the rarity of cases in the first and fifth categories. Each category of K^+^ concentration comprised 60 cases, and a total of 300 cases were used in the test. The participating physicians included an emergency physician under training (second-year resident); two emergency physicians, one with 4 and the other with 13 years of experience; a chief resident in cardiology; and two cardiologists, one with 2 and the other with 9 years of experience. The physicians had no access to patient information and no knowledge of the data. The responses they provided were entered into an online standardized data entry program. We calculated their sensitivity and specificity and compared their results with those of ECG12Net.

### Statistical Analysis and Model Performance Assessment

The study cohort was divided into training, validation, and test sets. We presented their characteristics as the means and standard deviations, the numbers of patients, or the percentages, where appropriate. This information was compared using either analysis of variance or the chi-square test as appropriate. We then analyzed the EMP differences between the five serum K^+^ groups, and the EMPs were subjected to post hoc analysis. All the dyskalemia groups were compared to the normal group.

The primary analysis was done to evaluate the performance in dyskalemia prediction between ECG12Net and the clinicians in a machine-human competition. Receiver operating characteristic curves and the areas under the curve (AUCs) were applied to evaluate the competition results. Additionally, the sensitivity, specificity, and balance accuracy of dyskalemia prediction by ECG12Net and the clinical physicians were calculated. The balance accuracy is defined as the mean of the sensitivity and specificity obtained in the study. Due to the stratified sampling process destroying the original prevalence, the positive predictive value and negative predictive value for the competition results are not presented.

The secondary analyses were performed on our test set with the data obtained after July 21, 2016, which had not been used in the training process. This was a simulated prospective study to evaluate the performance of the AI models with the mean absolute error (MAE) as the major measurement index due to the continuous predictions. Moreover, categorized analyses are also presented. Sensitivity, specificity, positive predictive value, negative predictive value, and the squared weighted kappa were used to evaluate the performance of the models. Finally, we conducted a series of logistic models to identify the effects of patient demographic characteristics on the performance of our deep-learning model.

We used a significance level of*P*< throughout the analysis. Bootstrap 95% CIs were calculated and presented for all measure indexes based on 10,000 permutations. No additional adjustments for multiple comparisons were used because of the small number of planned comparisons. The statistical analysis was carried out using the software environment R, version 3.4.3 (The R Foundation).

## Results

### Cohort Description

The training, validation, and test sets comprised records from 28,183; 3993; and 8004 patients, respectively. Table 1 shows the patient characteristics, which reveal similar distributions among the sets of gender, age, body mass index, marital status, education, and underlying comorbidities, including diabetes mellitus, coronary artery disease, hypertension, heart failure, hyperlipidemia, chronic kidney disease, chronic obstructive pulmonary disease, and pneumothorax. The training, validation, and test sets consisted of 46,692; 6407; and 13,222 pairs, respectively, of ECGs and K^+^ concentrations. The details of the laboratory and EMP analyses are presented in Multimedia Appendix 1. The detailed dyskalemia distribution (see Multimedia Appendix 1) shows a hypokalemia/hyperkalemia prevalence of 22.7%/2.6%, 22.9%/2.3%, and 22.7%/2.8% in the training, validation, and test sets, respectively.

**Table 1:** Patients’ characteristics in the training, validation, and test sets.

Characteristic			Training set (N=28,183)		Validation set (N=3993)		Test set (N=8004)		*P* value
**Gender, n (%)**									.08
	Female	13,828 (49.07)		1942 (48.64)		3814 (47.65)			
	Male	14,350 (50.92)		2049 (51.31)		4190 (52.35)			
Age (years), mean (SD)			62.57 (19.45)		62.47 (19.33)		62.61 (19.25)		.93
Height (cm), mean (SD)			162.24 (9.37)		162.19 (9.58)		163.29 (36.90)		.09
Weight (cm), mean (SD)			63.98 (14.12)		64.11 (14.16)		63.75 (13.79)		.78
BMI (kg/m^2^), mean (SD)			24.32 (6.38)		24.39 (6.71)		24.07 (4.49)		.24
**Underlying comorbidities, n (%)**									
	Diabetes mellitus	3553 (12.61)		476 (11.92)		1009 (12.61)		.47	
	Coronary artery disease	1694 (6.01)		257 (6.44)		485 (6.06)		.57	
	Hypertension	5219 (18.52)		741 (18.56)		1496 (18.69)		.94	
	Heart failure	825 (2.93)		124 (3.11)		239 (2.99)		.81	
	Hyperlipidemia	3868 (13.72)		520 (13.02)		1078 (13.47)		.45	
	Chronic kidney disease	6294 (22.33)		859 (21.51)		1786 (22.31)		.50	
	Chronic obstructive pulmonary disease	1351 (4.79)		193 (4.83)		408 (5.10)		.54	
	Pneumothorax	88 (0.31)		11 (0.28)		24 (0.30)		.92	

### Primary Analysis

**Figure 2:**
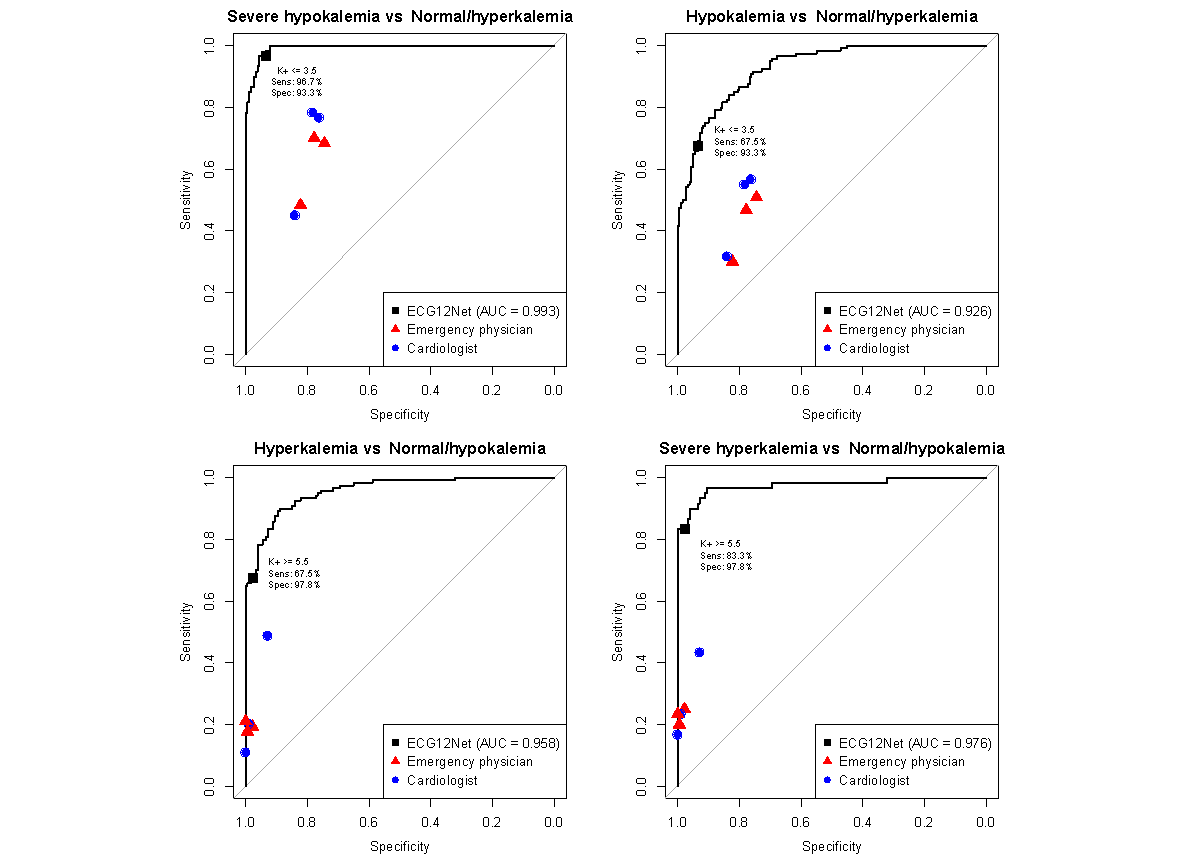
Performance comparison in detecting dyskalemias from the human-machine competition (n=300). The receiver operating characteristic curves are made by the predictions of ECG12Net-1. The red triangles and blue circles indicate emergency physicians and cardiologists, respectively, in the human-machine competition. K^+^ ≤2.5 mEq/L, 2.5< K^+^ ≤3.5 mEq/L, 3.5< K^+^ <5.5 mEq/L, 5.5≤ K^+^ <6.5 mEq/L, and K^+^ ≥6.5 mEq/L were defined as severe hypokalemia (n=60), hypokalemia (n=120), normal (n=60), hyperkalemia (n=120), and severe hyperkalemia (n=60), respectively. AUC: area under the curve.

The results of the human-machine competition are summarized in Figure 2. The AUCs of our ECG12Net-1 were 0.993, 0.926, 0.958, and 0.976 in the detection of severe hypokalemia, hypokalemia, hyperkalemia, and severe hyperkalemia, respectively. Due to the continuous nature of the K^+^ concentration predictions from ECG12Net, we used clinical cut points as described in the Methods section for further analysis. Our clinicians detected severe hypokalemia with sensitivities and specificities of 45%-78.3% and 74.4%-83.9%, respectively, whereas ECG12Net-1 achieved a sensitivity of 96.7% (95% CI 91.7-100.0) and a specificity of 93.3% (95% CI 89.4-96.7). In detecting severe hyperkalemia, the clinicians had nearly perfect specificity (92.8%-100.0%) but low sensitivity (16.7%-43.3%), while ECG12Net-1 exhibited a sensitivity of 83.3% (95% CI 73.3-91.7) and a specificity of 97.8% (95% CI 95.6-99.4). Including mild-to-moderate dyskalemias, ECG12Net-1 had the highest sensitivity in detecting hypokalemia (67.5%, 95% CI 59.2-75.8) and hyperkalemia (67.5%, 95% CI 59.2-75.8) in the human-machine competition. The details of the human-machine competition are shown in Table 2. In terms of balance accuracy, ECG12Net-1’s performance was significantly better than that of the best clinician (cardiologist 2) participating in the hypokalemia detection (80.4%, 95% CI 75.7-84.9, vs 66.7%, 95% CI 61.4-72.1). In detecting severe hyperkalemia, the balance accuracy of ECG12Net-1 was also significantly better than that of the best clinician (cardiologist 3) (82.7%, 95% CI 78.2-86.8, vs 70.6%, 95% CI 65.6-75.4). Although ECG12Net-2 exhibited lower performance compared with ECG12Net-1, it performed much better than all of the clinicians. The results of the consistency analysis are shown in Multimedia Appendix 1. When inconsistency arose between the predictions made by ECG12Net and the experts, ECG12Net was approximately 3.85 times more likely to be correct (*P*<.001 based on the McNemar test).

**Table 2:** Comparison between human experts and ECG12Net on the sensitivity and specificity in the subtest set (n=300).

Type of dyskalemia		Sensitivity^a^, 95% CI				Specificity^a^ (n=180), 95% CI		Balance accuracy^b^, 95% CI
		Overall (n=120)	Severe (n=60)	Mild to moderate (n=60)				
**Hypokalemia (K^+^≤3.5 mEq/L)**								
	Emergency physician 1^c^	0.300 (0.219-0.385)	0.483 (0.356-0.613)	0.117 (0.040-0.206)	0.822 (0.765-0.875)		0.561 (0.512-0.611)	
	Emergency physician 2^d^	0.508 (0.420-0.598)	0.683 (0.562-0.797)	0.333 (0.217-0.455)	0.744 (0.680-0.807)		0.626 (0.572-0.682)	
	Emergency physician 3^e^	0.467 (0.378-0.554)	0.700 (0.581-0.812)	0.233 (0.131-0.345)	0.778 (0.717-0.835)		0.622 (0.569-0.676)	
	Cardiologist 1^f^	0.317 (0.236-0.403)	0.450 (0.323-0.579)	0.183 (0.091-0.288)	0.839 (0.782-0.892)		0.578 (0.528-0.628)	
	Cardiologist 2^g^	0.550 (0.462-0.637)	0.783 (0.673-0.885)	0.317 (0.204-0.439)	0.783 (0.722-0.842)		0.667 (0.614-0.721)	
	Cardiologist 3^h^	0.567 (0.477-0.654)	0.767 (0.654-0.870)	0.367 (0.246-0.492)	0.761 (0.697-0.820)		0.664 (0.608-0.718)	
	ECG12Net-1	0.675 (0.592-0.758)	0.967 (0.917-1.000)	0.383 (0.267-0.500)	0.933 (0.894-0.967)		0.804 (0.757-0.849)	
	ECG12Net-2	0.675 (0.592-0.758)	0.967 (0.917-1.000)	0.383 (0.267-0.500)	0.922 (0.883-0.961)		0.799 (0.751-0.843)	
**Hyperkalemia (K^+^≥5.5 mEq/L)**								
	Emergency physician 1	0.192 (0.124-0.266)	0.250 (0.145-0.365)	0.133 (0.053-0.224)	0.978 (0.954-0.995)		0.585 (0.549-0.623)	
	Emergency physician 2	0.175 (0.110-0.244)	0.200 (0.103-0.304)	0.150 (0.065-0.250)	0.994 (0.982-1.000)		0.585 (0.552-0.620)	
	Emergency physician 3	0.208 (0.137-0.282)	0.233 (0.130-0.344)	0.183 (0.089-0.288)	1.000 (1.000-1.000)		0.604 (0.569-0.641)	
	Cardiologist 1	0.108 (0.056-0.167)	0.167 (0.077-0.266)	0.050 (0.000-0.113)	1.000 (1.000-1.000)		0.554 (0.528-0.583)	
	Cardiologist 2	0.200 (0.131-0.274)	0.233 (0.132-0.345)	0.167 (0.078-0.265)	0.989 (0.971-1.000)		0.594 (0.560-0.632)	
	Cardiologist 3	0.483 (0.393-0.571)	0.433 (0.305-0.558)	0.533 (0.403-0.661)	0.928 (0.888-0.963)		0.706 (0.656-0.754)	
	ECG12Net-1	0.675 (0.592-0.758)	0.833 (0.733-0.917)	0.517 (0.383-0.633)	0.978 (0.956-0.994)		0.827 (0.782-0.868)	
	ECG12Net-2	0.683 (0.600-0.767)	0.833 (0.733-0.917)	0.533 (0.400-0.650)	0.972 (0.944-0.994)		0.828 (0.783-0.869)	

^a^The test provides three selections for prediction: hypokalemia (K^+^ ≤3.5 mEq/L), normokalemia (3.5 mEq/L< K^+^ <5.5 mEq/L), and hyperkalemia (K^+^ ≥5.5 mEq/L).

^b^The balance accuracy value represents the average of the overall sensitivity and specificity.

^c^Emergency physician 1: second-year resident.

^d^Emergency physician 2: 4 years of experience.

^e^Emergency physician 3: 13 years of experience.

^f^Cardiologist 1: chief resident of cardiology.

^g^Cardiologist 2: 2 years of experience.

^h^Cardiologist 3: 9 years of experience.

### Performance of ECG12Net on the Test Set

**Figure 3:**
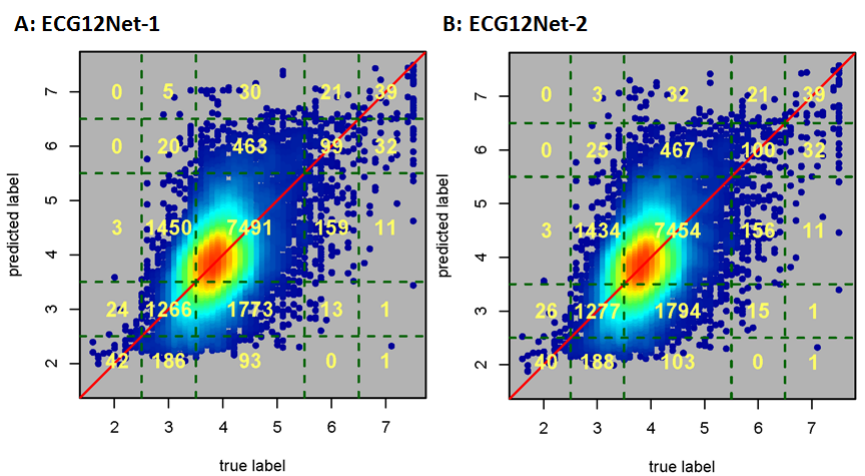
Confusion scatter plots of ECG12Net-1 and ECG12Net-2 predictions on the test set (n=13,222). The x-axis indicates the true K+ concentration from laboratory testing. The y-axis presents the predicted K+ concentration by ECG12Net-1 (A) and ECG12Net-2 (B). Red points represent the highest density, followed by yellow, green, light blue, and dark blue. Perfect model performance would fall only along the red diagonal line. We categorized the K+ concentration into five groups (K^+^ ≤2.5 mEq/L, 2.5< K^+^ ≤ 3.5 mEq/L, 3.5< K^+^ <5.5 mEq/L, 5.5≤ K^+^ <6.5 mEq/L, and K^+^ ≥6.5 mEq/L) and calculated the case counts in each grid.

The model performance on the test set is shown in Multimedia Appendix 1. The performance of ECG12Net was better than that of each lead. ECG12Net-1 had the lowest MAE (0.531). Including EMP information did not improve the prediction of K^+^ concentration (MAE ECG12Net-1: 0.531; MAE ECG12Net-2: 0.538). When categorizing among three classes—hypokalemia, normokalemia, and hyperkalemia—and five classes, with the addition of severe hypokalemia and severe hyperkalemia, as described in Multimedia Appendix 1, a similar performance was observed by ECG12Net-1; this demonstrated the highest squared weighted kappa of 0.354 in the three-class categorization and 0.396 in the five-class categorization. For the detection of hypokalemia, the sensitivity, specificity, positive predictive value, and negative predictive value of ECG12Net-1 were 50.7%, 81.6%, 44.7%, and 85.0%, respectively; for hyperkalemia, they were 50.8%, 96.0%, 26.9%, and 98.5%, respectively. The confusion scatter plots for the predictions by the two ECG12Nets are shown in Figure 3. Importantly, in detecting severe hypokalemia and hyperkalemia, ECG12Net-1 demonstrated a sensitivity of 95.6% and 84.5%, respectively. ECG12Net-2 exhibited similar prediction capabilities for severe hypokalemia and hyperkalemia as ECG12Net-1.

### Model Interpretation

**Figure 4:**
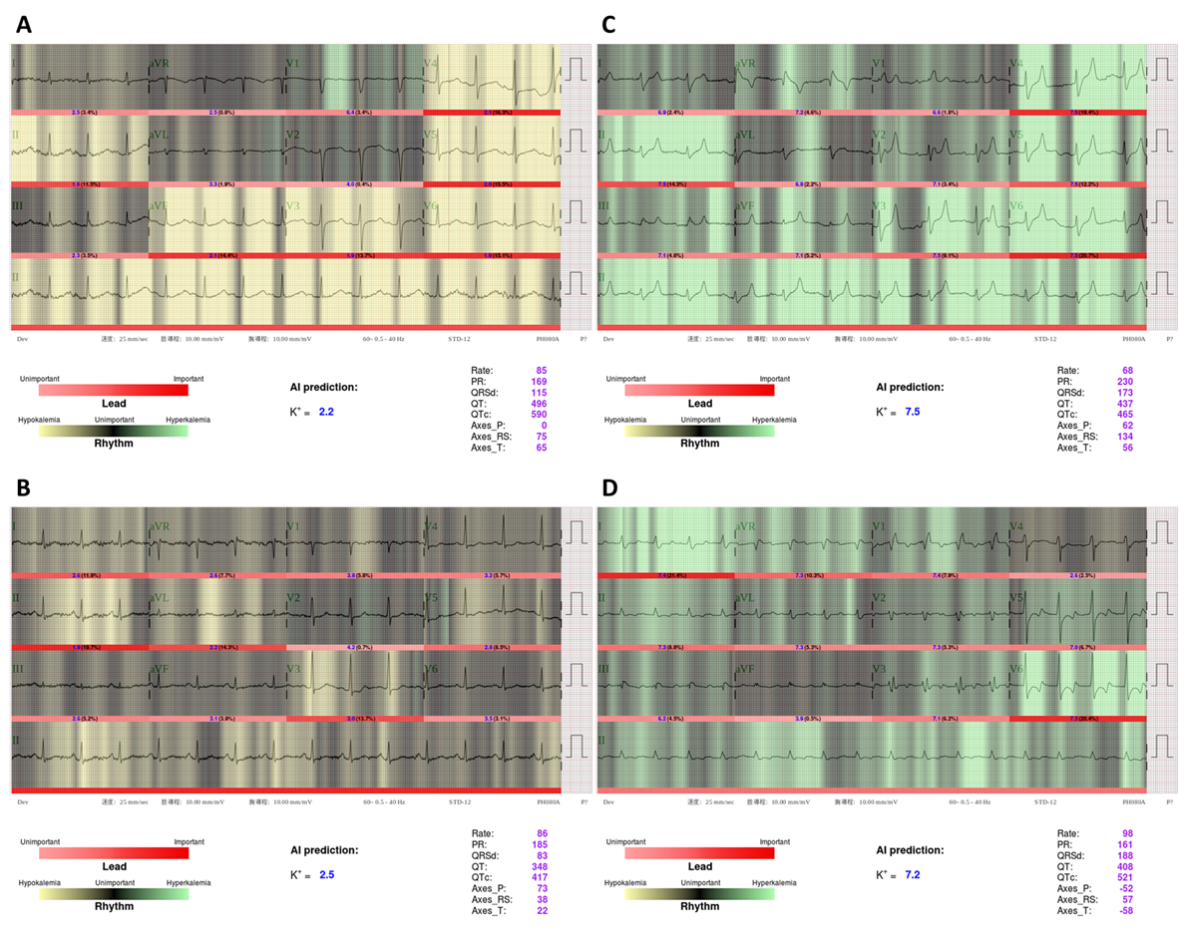
Visualization analysis for ECG12Net-1 in selected severe hypokalemia and hyperkalemia cases. The lighter areas (green or yellow) indicate areas of focus by ECG12Net-1. Clinicians consistently recognized panel A as a typical case of severe hypokalemia but overlooked panel B. Similarly, clinicians consistently recognized panel C as severe hyperkalemia but overlooked panel D. From A to D, the real K^+^ concentrations were 2.3 mEq/L, 2.5 mEq/L, 9.1 mEq/L, and 7.1 mEq/L, respectively. AI: artificial intelligence.

A total of 58 severe hypokalemia cases were correctly detected by ECG12Net-1, of which 15 (26%) were overlooked by clinician consensus. The classical ECG findings of U wave and ST segment depression, especially in leads V2 and V3, were consistently recognized as severe hypokalemia by both the clinicians and ECG12Net-1 (see Figure 4 A). As shown in Figure 4 B, ECG12Net-1 predicted a case of severe hypokalemia from ST segment depression in the V3 lead; this case was misdiagnosed by all the clinicians. Two cases of severe hypokalemia were misclassified by ECG12Net-1 but diagnosed correctly by the clinicians (data not shown). These cases had severe noise in the presented ECG; however, the clinicians made the correct diagnosis based on the presence of a prolonged QTc.

A total of 50 severe hyperkalemia cases were correctly detected by ECG12Net-1, with 36 (72%) of these cases overlooked by clinician consensus. Figure 4 C shows a typical ECG presentation of severe hyperkalemia with tented T waves accompanied by a long QRS complex duration, which was correctly diagnosed by all clinicians and ECG12Net-1. Figure 4 D shows a case of severe hyperkalemia correctly recognized by ECG12Net-1, with ST depression followed by a peaked T wave in lead V6, which was misdiagnosed as hypokalemia by all the clinicians. There were also 10 cases of severe hyperkalemia overlooked by ECG12Net-1 and all clinicians.

## Discussion

In this study, we developed a deep-learning model, ECG12Net, to detect dyskalemias through ECG analysis. Using a deep convolutional network extracting many useful ECG features with a training set of more than 50,000 ECGs, ECG12Net performed better than clinicians in detecting dyskalemias. Notably, ECG12Net performed well with sensitivities of 95.6% and 84.5% in detecting severe hypokalemia and severe hyperkalemia, respectively.

ECG interpretation is one of the most important skills in medical practice. Previous studies have analyzed morphological features, for instance, the R wave peak [26] and the QRS complex [27], combined with machine learning approaches for disease detection, such as atrial fibrillation [28]. These systems were relatively imprecise, making it troublesome to quantify specific rhythm morphologies [29]. Although some recent studies have used deep convolutional neural networks and recurrent neural networks mainly for arrhythmia detection [30-35], most of the data were collected from wearable devices without offering all the important information provided by a 12-lead ECG [11]. The clinical value of these findings is also dampened by the lack of laboratory-based diagnosis and annotation and the relatively small volumes of data. In contrast, our database was unprecedented, comprising 40,180 patients and 66,321 laboratory-annotated ECG records collected by standard 12-lead ECG machines.

Galloway et al recently developed a deep-learning model to screen for hyperkalemia in patients with chronic kidney disease, stage III or higher, using ECG [36]. We applied ECG12Net to a broad set of patients in the ED and developed a continuous prediction of both hypokalemia and hyperkalemia. Moreover, although the three-category classification task in our study is more difficult than the two-category classification task in theirs, our ECG12Net achieved an AUC greater than 0.9 in detecting hyperkalemia, which is similar to that of their model with an AUC of 0.85-0.88. This highlights the strength of ECG12Net.

The EMPs of different K^+^ concentration groups yielded several interesting findings. The EMPs, such as the PR and QTc intervals, and the data used for analysis were all collected from the original ECGs (see Multimedia Appendix 1). The impact of hyperkalemia on the T wave axis was more profound and substantial than the axes of the P and RS waves. Hypokalemia was actually associated with a widening of the QRS complex, which may be explained by the decrease in conduction velocity caused by reduced K^+^ concentrations after hemodialysis [37]. Although the longest QTc occurred in the severe hypokalemia group, a well-documented finding, the QTc was longer in patients with hyperkalemia as well. In fact, for most of the intervals and durations, the nadir was in normokalemia, with increases on both forms of dyskalemia. Although the underlying mechanisms are unclear, these findings uncovered by big data may guide directions for further research.

Interestingly, the algorithm focusing only on morphologic changes (ie, ECG12Net-1) performed slightly better than that with additional EMP information (ie, ECG12Net-2). That the addition of EMP information did not improve the model’s predictive ability corroborates prior research that found that deep-learning models can automatically extract useful features for prediction without preprocessing [17,20,21]. This also highlights the importance of morphologic changes in ECG over EMPs in the detection of dyskalemias.

There are several clinical applications of ECG12Net shown in Multimedia Appendix 1. First, severe dyskalemia could be identified by ECG12Net within 5 minutes, much faster than laboratory testing, leading to more prompt management. Second, pseudodyskalemia, defined as an abnormal reported serum or plasma K^+^ concentration despite a normal in vivo K^+^ concentration, can be excluded early by ECG12Net to avoid inappropriate treatment. Third, the performance of ECG12Net is more than 10% better than that of the best cardiologist in our study, whose performance was similar to other experts in prior studies [38,39]. This means that emergency physicians could have access to a consistent, beyond cardiologist-level decision aid available 24 hours a day to help diagnose and manage dyskalemic patients. Fourth, the developed ECG12Net model can be included in a wearable device for dyskalemia detection, especially for patients with advanced chronic kidney disease or uremia on dialysis. Finally, the ECG12Net model could be incorporated into ECG machines in ambulances or remote areas to facilitate telemedicine.

Explainable AI plays a critical role in clinical practice [40,41]. The so-called “black box” approach in the deep-learning models often precludes the understanding of the decision-making process [42]. To increase the interpretability of our model, we established heatmaps to visualize the focus in the ECG by ECG12Net using class activation mappings [25,43], which can help physicians understand the logic of the AI decisions. Although our ECG12Net was approximately 3.85 times more likely to be correct when inconsistencies occurred between the AI and human predictions (see Multimedia Appendix 1), physicians who can integrate the AI suggestions with the symptoms and signs of patients should make the final decision to take appropriate action.

Some limitations of this study should be mentioned. First, the studied patients were only enrolled from one academic medical center, despite the similar distribution of blood K^+^ concentration in other large studies [44,45]. Multicenter validation is needed to confirm the value and application of this study. Second, only six clinicians participated in the competition with ECG12Net’s performance. Although their performance in severe hyperkalemia detection was consistent with that of the previous studies [38,39], comparisons should be made with more experts to confirm the superiority of ECG12Net. Third, only the patients in the ED with both an ECG and a serum K^+^ test were enrolled in this study, which may have caused selection bias and constrained the generalizability of the results. Fourth, although the sensitivity heatmap provides a glimpse into the basis for ECG12Net’s prediction, the reason why the particular ECG segment was highlighted remains unclear. Finally, ECG12Net showed decreased sensitivity in detecting mild-to-moderate hypokalemia, which accounts for the majority of dyskalemias, leading to low *weighted averages* of the sensitivities. Hypokalemia-associated ECG changes usually occur when the serum K^+^ level falls below 3 mEq/L [46], which may explain why our algorithm failed to accurately distinguish the ECG morphologies of mild-to-moderate hypokalemia from normokalemia.

In conclusion, we established a deep-learning model called ECG12Net to detect dyskalemias in the ED. The collaboration between physicians and AI can lead to better health care for our patients. This model will help emergency physicians promptly recognize severe dyskalemias and potentially reduce sudden cardiac death.

## Appendix

Multimedia Appendix 1 Supplementary materials.

## Abbreviations

AI: artificial intelligence
AUC: area under the curve
ECG: electrocardiography
ED: emergency department
EMP: electrocardiography morphology parameter
IRB: Institutional Review Board
ISO: International Organization for Standardization
MAE: mean absolute error
QTc: corrected QT interval
