# Supplementary material for: A Deep-Learning Algorithm (ECG12Net) for Detecting Hypokalemia and Hyperkalemia by Electrocardiography: Algorithm Development
Source: JMIR Med Inform. 2020 Mar 5;8(3):e15931. doi: 10.2196/15931 (PMC7082733; doi:10.2196/15931)
Supplement: Multimedia Appendix 1 [file medinform_v8i3e15931_app1.docx]

**A Deep Learning Algorithm (ECG12Net) for Detecting Hypokalemia and Hyperkalemia by Electrocardiography: Algorithm Development**

**Supplementary Materials**

**Implementation details of ECG12Net**

***ECG12Net architecture***

Suppose that a standard 12-lead ECG signal comprises 12 sequences of N numbers (*N* = 1,250 in our database). To detect ECG-related serum K^+^ concentrations, ECG signal sequence *X* = [*x*_1,1_, *x*_1,2_, …, *x*_1,N_; *x*_2,1_, *x*_2,2_, …, *x*_2,N_; …; *x*_12,1_, *x*_12,2_, …, *x*_12,N_] is used as the input, and the output is a continuous K^+^ concentration ranging from 1.5 mEq/L to 7.5 mEq/L. Each output label corresponds to a segment of the input. Because the ECG information is mostly provided by morphologic changes with shift invariance, convolutional layers with weight sharing were used to adapt to this situation and reduce the risk of overfitting. We therefore developed a 12-channel sequence-to-sequence model to conduct this task as a revision of DenseNet ^1^. The complete architecture of ECG12Net is shown in Fig 1 in the main article.

A numeral sequence of length 1,024 was used as the input of each ECG lead in our experiment. However, the original data shape of our ECG lead is a vector of length 1,250, thus the original ECG lead data were randomly cropped to a length of 1,024 sequences as input in the training process. During the application, the first 1,024 numeral sequences and the last 1,024 numeral sequences were defined as the input data. The average value from the predictions was the final output (for the validation and test sets). Each lead input passes through the “ECG lead block”, and useful features were extracted by each “ECG lead block”, which share the same parameters. The ECG lead block consists of 80 trainable layers, as shown in Fig1A. The input data (1,024×1×1) first passes through a batch normalization layer (BN), followed by 32 convolutional filters with a 7×1 kernel size and 2×1 stride, another BN ^2^, and a rectified linear unit layer (ReLU) ^3^. Therefore, the data shape is 512×1×32, which is then used as input to the loop modules.

There are two modules used in this study. The first is the “dense block”. We defined a “dense unit” as a neural combination as follows: (1) a 1×1 convolution layer (stride = 1×1) with 128 filters to reduce the dimensions of the data, (2) a BN to normalize the input data, (3) a ReLU for nonlinearization, (4) a 3×1 convolution layer (stride = *k*×1) with 128 filters to extract features, (5) a BN for normalization, (6) a ReLU for nonlinearization, (7) a 1×1 convolution layer (stride = 1×1) with 32 filters, (8) a BN for normalization, (9) a ReLU for nonlinearization to extract features. After using a dense unit to extract features, we use dense connectivity, which provides direct connections from any layer to all subsequent layers, to build a “dense block.” The ECG lead block includes five dense blocks. Each comprises, in order, 3, 3, 6, 6, and 3 dense units. The stride parameter *k* was set to 1 to preserve the data shape in the dense block.

Dense blocks cannot be concatenated when the data shape changes. A pooling block was used to concatenate each dense block for downsampling in our architecture. This pooling block includes a dense unit (with the stride parameter *k* set to 2) and an average pooling layer (pool) with a 2×1 kernel size (stride = 2×1) ^4^. Each dense block is concatenated by the pooling block to integrate the features of the previous blocks.

The initial input shape before entering the loop modules is 512×1×32. The input passes through a pooling block, and its shape is changed to 256×1×64, followed by a dense block with 3 dense units (output shape = 256×1×160), a pooling block (output shape = 128×1×192), a dense block with 3 dense units (output shape = 128×1×288), a pooling block (output shape = 64×1×320), a dense block with 6 dense units (output shape = 64×1×512), a pooling block (output shape = 32×1×544), a dense block with 6 dense units (output shape = 32×1×736), a pooling block (output shape = 16×1×768), and a dense block with 3 dense units (output shape = 16×1×864). After the last dense block, the input passes through a BN (output shape = 16×1×864), a ReLU (output shape = 16×1×864), and a global pool (output shape = 1×1×864). Finally, a fully connected layer (FC) with 1 output was created (output shape = 1×1×1). This ECG lead block was used to extract 864 features from each ECG lead, making a basic output prediction. Except for the last FC, all model weights were the same in the 12 ECG lead blocks.

Fig. 1-B shows how ECG12Net integrates all the information from the ECG leads to make an overall prediction. ECG12Net comprises 12 ECG lead blocks corresponding to the lead sequences. An attention mechanism was designed based on a hierarchical attention network to concatenate these blocks and increase the interpretive power of ECG12Net ^5^. The attention block comprises an FC, a BN, a ReLU, an FC, and a BN. The first and second FCs contain 8 and 1 neurons, respectively. Attention scores are calculated from each ECG lead, which are integrated for standardization by a linear output layer. The standardized attention scores are used to weight the 12 ECG lead outputs by simple multiplication. The 12 weighted outputs are summed and converted into a linear output layer to generate the final prediction value. The above model using ECG information only was named ECG12Net-1, which contains 82 trainable layers. To improve prediction performance, we added an EMPNet, a multilayer perceptron with two hidden layers of 32 and 16 neurons and eight EMPs, to ECG12Net-1 to create ECG12Net-2. To prevent the potential poor performance from a shallow multilayer perceptron, we used a support vector machine, a random forest, and an eXtreme Gradient Boosting model for baseline model comparisons. A dropout layer ^6^ was added to the last FC, and the dropout rate was set at 0.5.

To increase the nonlinear adaptability and reduce the predicted monotonic linear function of output, we used categorywise label encoding technology to code the K^+^ concentration ^7^. Because the range of K^+^ concentrations is defined from 1.5 mEq/L to 7.5 mEq/L, we designed a 60-point sigmoid output with an interval of 0 to 1. For example, the minimal K^+^ concentration category of less than 1.5 mEq/L is coded as [0, 0, 0, 0, 0, 0, 0, 0, 0, 0, 0, 0, 0, 0, 0, 0, 0, 0, 0, 0, 0, 0, 0, 0, 0, 0, 0, 0, 0, 0, 0, 0, 0, 0, 0, 0, 0, 0, 0, 0, 0, 0, 0, 0, 0, 0, 0, 0, 0, 0, 0, 0, 0, 0, 0, 0, 0, 0, 0, 0], the K^+^ concentration category of 4.0 mEq/L is coded as [1, 1, 1, 1, 1, 1, 1, 1, 1, 1, 1, 1, 1, 1, 1, 1, 1, 1, 1, 1, 1, 1, 1, 1, 1, 0, 0, 0, 0, 0, 0, 0, 0, 0, 0, 0, 0, 0, 0, 0, 0, 0, 0, 0, 0, 0, 0, 0, 0, 0, 0, 0, 0, 0, 0, 0, 0, 0, 0, 0], the K^+^ concentration category of 7.3 mEq/L is coded as [1, 1, 1, 1, 1, 1, 1, 1, 1, 1, 1, 1, 1, 1, 1, 1, 1, 1, 1, 1, 1, 1, 1, 1, 1, 1, 1, 1, 1, 1, 1, 1, 1, 1, 1, 1, 1, 1, 1, 1, 1, 1, 1, 1, 1, 1, 1, 1, 1, 1, 1, 1, 1, 1, 1, 1, 1, 1, 0, 0], and so on. A cross-entropy loss function is used in these sigmoid outputs, and our network was trained to minimize the cross-entropy loss. The final prediction is the sum of these values divided by 10 plus 1.5. For example, one prediction vector given by ECG12Net is [1, 1, 1, 1, 1, 1, 1, 1, 1, 0.9, 0.8, 0.7, 0, 0, 0, 0, 0, 0, 0, 0, 0, 0, 0, 0, 0, 0, 0, 0, 0, 0, 0, 0, 0, 0, 0, 0, 0, 0, 0, 0, 0, 0, 0, 0, 0, 0, 0, 0, 0, 0, 0, 0, 0, 0, 0, 0, 0, 0, 0, 0], which corresponds to a K^+^ concentration of 2.64 mEq/L.

***Training details***

The 12-lead ECG signal sequences were first trained separately by the K^+^ concentration data from each lead. Due to the significantly uneven distribution of K^+^ concentrations in our study, an oversampling process was implemented to ensure that rare samples were adequately recognized ^8, 9^. The sample weights were the multiplicative inverse of the 5-class frequencies in the training set. In our experiments, the sample sizes of each K^+^ concentration group (K^+^ ≤ 2.5, 2.5 < K^+^ ≤ 3.5, 3.5 < K^+^ < 5.5, 5.5 ≤ K^+^ < 6.5, and 6.5 ≤ K^+^) were 207 (0.4%), 10394 (22.3%), 34854 (74.6%), 954 (2.0%), and 283 (0.6%), respectively. For example, the sample weights of an ECG signal with a K^+^ concentration of 2.3/3.7/5.3/6.2 will be 255.6/1.3/1.3/48.9, respectively. The mini-batch samples were sampled based on these weights, which sufficiently considered rare dyskalemia cases and was not be skewed by the overwhelming number of normal cases. The software package MXNet version 1.2.0 ^10^ was implemented in ECG12Net. The settings for the training model were as follows: (1) an Adam optimizer with standard parameters (β_1_ = 0.9 and β_2_ = 0.999) and a batch size of 50 for optimization; (2) an initial learning rate set to 0.001 and lowered by 10 three times when validation loss plateaued after an epoch; and (3) a weight decay of 10^−4^ ^11^. The model with the highest validation performance was selected as the final model, which presented performance in the test set only evaluated once.

***Data augmentation***

A previous study reported severe overfitting in an atrial fibrillation detection task and suggested a series of data augmentations to improve model performance ^12^. In the current study, the problem of overfitting was due to the large number of parameters in the deep learning architecture (~3 million trainable parameters) relative to the sample size. The first step in tackling this issue was to resize the sequence length by adjusting the heart rate. We randomly resampled a broad range of heart rates in a uniform distribution from 0.8*HR* to 1.2*HR*, where *HR* is the original heart rate for each sample. The second step was to add a random variable drawn from a Gaussian distribution with a mean of 0 and a standard deviation of 0.1. Third, time points were selected uniformly and at random, and the ECG signal values in a 50 ms vicinity of these points were set at 0. This method is called dropout burst ^12^. Finally, we set six random ECG lead sequences to 0 in the combined training step. Our preliminary experiment indicated that the initial model made a perfect prediction based on information from only a few ECG leads (V3, V4, and lead II) in the training set. However, the model performed poorly in the validation set due to having stopped learning features from other ECG leads. Therefore, we set six random ECG lead sequences to 0 in the training step, which forced the model to learn from all the leads.

***Model visualization***

To interpret the network predictions, we created heatmaps to visualize the ECG rhythms and leads using class activation mappings (CAMs) ^13^ and attention mechanisms ^5^. This was based on the global average pooling (GAP) architecture in the last network, which was used at the end of each ECG lead. In addition, the various contributions made to the final prediction by each ECG lead were weighted by the attention mechanisms, which were used to visualize the importance of each ECG lead.

**Details of the cohort description**

***Laboratory analysis***

The laboratory results of the above 66,321 emergency records classified by K^+^ levels are shown in Supplementary Table MA1-1. The severe hypokalemia (K^+^ ≤ 2.5 mEq/L), mild to moderate hypokalemia (2.5 < K^+^ ≤ 3.5 mEq/L), normal (3.5 < K^+^ < 5.5 mEq/L), mild to moderate hyperkalemia (5.5 ≤ K^+^ < 6.5 mEq/L), and severe hyperkalemia (6.5 ≤ K^+^) groups comprised 301, 14,765, 49,496, 1,367, and 392 emergency records, respectively. K^+^ concentration was significantly associated with most laboratory results except the levels of HbA1c and troponin I. As indicated in Supplementary Table MA1-1, poor kidney function indexes (blood urea nitrogen, creatinine, and GFR) are highly represented in the severe hyperkalemia group, followed by the mild to moderate hyperkalemia group.

***EMP analysis***

Supplementary Table MA1-2 shows the EMPs for the ECG records of the different K^+^ concentration groups. Overall, the records for the different K^+^ concentration groups were evenly distributed among the training set, the validation set, and the test set. Patients with all types of dyskalemia except severe hyperkalemia showed a significantly increased heart rate. We also observed a significant increase in the PR interval in the patients with hyperkalemia. Additionally, the QRS duration and QTc were significantly increased in patients with dyskalemia. The trends in the axes for other K^+^ concentration groups were less obvious. Post hoc analysis revealed significant signals in the RS wave axes and the T wave axes, but no dose response effect was observed. The large number of significant signals revealed that EMP may contribute some predictive capacities in the serum K^+^ concentration detection task.

**Human-machine competition**

***Consistency analysis***

The results of the consistency analysis are shown in Supplementary Fig 1. Clustering analysis revealed a higher consistency between the experts, especially the board-certified physicians (emergency physician 2, emergency physician 3, cardiologist 2, and cardiologist 3), than the two ECG12Nets. In the pair analysis, an expert consensus was generated from the votes of the six physicians. A consensus was considered to have been reached if the experts offered four consistent votes. If three or fewer votes were consistent, the consensus depended primarily on the consistency of the opinions of the cardiologists. The results for ECG12Net-1 were consistent with the expert consensus for 169 records. One hundred and four (61.5%) of these predictions were correct. Of the 131 records in which the results of ECG12Net-1 were inconsistent with the expert consensus, 104 (79.4%) were won by ECG12Net-1. When inconsistency arose between the predictions made by ECG12Net and the experts, ECG12Net was approximately 3.85 times more likely to be correct (p < 0.0001 based on McNemar’s test). ECG12Net-2 performed slightly less well than ECG12Net-1 in this competition.

**Analysis with the whole test set**

***Performance of ECG12Net***

The MAE and other categorized analyses are shown in Supplementary Table MA1-3. The predictive capacity was evaluated by the output of each ECG lead block. We found that lead V5 provided the lowest MAE (0.742), followed by V6 (0.748), V3 (0.775), lead II (0.784), etc. ECG12Net-1, a deep learning model, makes predictions based solely on ECG image data, which integrates all the information from the 12 ECG leads and weights them by an attention mechanism. ECG12Net-1 had a much better MAE (0.531) than the best ECG lead block, V5 (0.742), which highlights the importance of integrating the information from all 12 leads. There were four versions of EMPNet tested for estimating K^+^ concentration in which the MAEs were significantly higher than those of ECG12Net-1 (0.699/0.565/0.603/0.605 vs. 0.531). ECG12Net-2 integrated ECG12Net-1 and the multilayer perceptron version of EMPNet. Due to non-differentiability, the versions with the support vector machine, random forest, or eXtreme Gradient Boosting model were not applied. Intriguingly, extra EMP information could not increase the performance in estimating K^+^ concentration (ECG12Net-1: 0.531→ECG12Net-2: 0.538).

Because physicians often divide serum potassium abnormalities into three categories in clinical practice, we also presented the categorized analysis for comparing model performance. ECG12Net-1 showed a sensitivity of 50.7%/50.8%, a specificity of 81.6%/96.0%, and a positive predictive value of 44.7%/26.9% for hypokalemia and hyperkalemia, respectively. The 3-class and 5-class squared weighted kappa values were the global performance index in our categorized analysis: ECG12Net-1 shows the highest values of 0.354 and 0.396, respectively, compared with other models. The performance ranking between leads was slightly altered. V3 and V6 had the highest 3-class (0.239) and 5-class (0.261) kappa values, respectively. The EMPNets show the worst performance in this categorized analysis. This is due to a lack of information for prediction, so the two EMPNets often give mean predictions. A model that produces mean predictions often has a relatively low MAE, but this indicates that it has no predictive ability. In summary, the 12-lead information is very important, and extra EMPs cannot provide additional performance improvement.

***Effect of different patient characteristics***

The patients recruited in the study were only from the emergency department (ED) in an overwhelmingly Chinese population. Previous studies have demonstrated some subtle differences in ECG features among women of different ethnicities but not among men^14^. Therefore, ECG12Net should be tested in a target population before widespread use. The baseline ECG changes might be confounded by gender, body mass index (BMI), and comorbidities, such as chronic obstructive pulmonary disease (COPD). A series of experiments was conducted to evaluate the effect of these patient factors on K+ concentration prediction, as shown in Supplementary Fig 2. The variables used in the logistic models were gender, age, BMI, and all comorbidities, including diabetes, coronary artery disease, hypertension, heart failure, hyperlipidemia, CKD, COPD, and pneumothorax. The logistic model using patient characteristics only showed worse performance than ECG12Net-1. Interestingly, compared with ECG12Net-1, the combination model, which combined patient characteristics and ECG12Net-1, had almost similar performance in the detection of hypokalemia and severe hypokalemia (0.979/0.774 vs. 0.976/0.750), and in the detection of hyperkalemia and severe hyperkalemia (0.908/0.973 vs. 0.911/0.974). Such variable performances between ECG12Net-1 and the combination model indicate that the comorbidities might not have confounding effects on the predictions in this study. Although the effects of rare circumstances, such drug overdose, on the model performance were not evaluated in current study, the detection of dyskalemia by ECG12Net may be practical to populations other than ED patients.

**Supplementary Table MA1-1. Pertinent laboratory results for different serum K+ classification groups.**

|  | **Serum K^+^ concentration** | | | | | **p-value** |
| --- | --- | --- | --- | --- | --- | --- |
|  | **K^+^ ≤ 2.5**  **(n = 301)** | **2.5 < K^+^ ≤ 3.5**  **(n = 14,765)** | **3.5 < K^+^ < 5.5**  **(n = 49,496)** | **5.5 ≤ K^+^ < 6.5**  **(n = 1,367)** | **6.5 ≤ K^+^**  **(n = 392)** |  |
| **K^+^ (mEq/L)** | 2.3±0.2 | 3.3±0.2 | 4.1±0.4 | 5.8±0.3 | 7.2±0.7 |  |
| **Na^+^ (mEq/L)** | 134.9±8.0 | 136.7±5.0 | 136.2±5.1 | 133.9±6.8 | 133.8±7.4 | <0.0001 |
| **Cl^-^ (mEq/L)** | 99.0±10.9 | 102.0±6.3 | 102.3±6.1 | 101.1±8.7 | 101.3±9.4 | <0.0001 |
| **TCa^++^ (mg/dL)** | 8.4±1.0 | 8.6±0.7 | 8.6±0.7 | 8.7±0.9 | 8.5±1.0 | <0.0001 |
| **Mg^++^ (mg/dL)** | 1.9±0.5 | 2.0±0.4 | 2.1±0.4 | 2.3±0.5 | 2.4±0.6 | <0.0001 |
| **Hb (g/dL)** | 12.0±2.5 | 12.7±2.3 | 12.7±2.4 | 10.7±2.6 | 10.7±2.9 | <0.0001 |
| **GLU (mg/dL)** | 165.1±123.5 | 141.3±72.1 | 146.6±83.7 | 180.4±152.3 | 193.5±200.8 | <0.0001 |
| **HbA1c (%)** | 6.8±2.3 | 7.3±2.3 | 7.4±2.3 | 7.2±2.0 | 7.3±2.1 | 0.0539 |
| **ALT (U/L)** | 46.7±253.8 | 34.3±101.2 | 35.0±110.3 | 55.4±190.7 | 47.5±139.7 | <0.0001 |
| **CK (U/L)** | 407.8±1146.1 | 167.4±518.4 | 156.7±492.9 | 259.0±856.6 | 305.5±784.4 | <0.0001 |
| **Tro I (ng/mL)** | 0.3±1.2 | 0.5±4.0 | 0.4±3.1 | 0.6±3.3 | 0.6±3.0 | 0.3514 |
| **BUN (mg/dL)** | 28.4±26.5 | 22.4±20.1 | 27.3±24.3 | 57.5±42.1 | 67.3±52.2 | <0.0001 |
| **Cr (mg/dL)** | 1.6±1.7 | 1.3±1.5 | 1.5±2.0 | 4.2±3.9 | 4.7±4.9 | <0.0001 |
| **GFR**  **(ml/min/1.73 m^2^)** | 70.6±39.6 | 76.8±31.4 | 70.6±32.9 | 31.3±30.0 | 29.1±28.9 | <0.0001 |

K^+^, Potassium; Na^+^, Sodium; Cl^-^, Chloride; TCa^++^, total calcium; Mg^++^, magnesium; Hb, hemoglobin; GLU, glucose at ER; HbA1c, hemoglobin A1c; ALT, alanine aminotransferase; CK, creatine kinase; Tro I, troponin I; BUN, blood urea nitrogen; Cr, creatinine; GFR, glomerular filtration rate. The results of significant post hoc analyses (Bonferroni method) are as follows: K^+^ ≤ 2.5 vs. 3.5 < K^+^ < 5.5: Na^+^, Cl^-^, Ca^++^, Mg^++^, Hb, GLU, and CK; 2.5 < K^+^ ≤ 3.5 vs. 3.5 <K^+^ < 5.5: Na^+^, Cl^-^, Ca^++^, Mg^++^, GLU, BUN, Cr, and GFR; 5.5 ≤ K^+^ < 6.5 vs. 3.5 <K^+^ < 5.5: Na^+^, Cl^-^, Ca^++^, Mg^++^, Hb, GLU, ALT, CK, BUN, Cr, and GFR; 6.5 ≤ K^+^ vs. 3.5 < K^+^ < 5.5: Na^+^, Cl^-^, Mg^++^, Hb, GLU, CK, BUN, Cr, and GFR.

**Supplementary Table MA1-2. ECG morphology parameters (EMP) in the different serum K^+^ classification groups.**

|  | **Serum K^+^ concentration** | | | | | **p-value** |
| --- | --- | --- | --- | --- | --- | --- |
|  | **K^+^ ≤ 2.5**  **(n = 301)** | **2.5 < K^+^ ≤ 3.5**  **(n = 14,765)** | **3.5 < K^+^ < 5.5**  **(n = 49,496)** | **5.5 ≤ K^+^ < 6.5**  **(n = 1,367)** | **6.5 ≤ K^+^**  **(n = 392)** |  |
| **Dataset** |  |  |  |  |  | 0.2807 |
| Training set | 207(68.8%) | 10394(70.4%) | 34854(70.4%) | 954(69.8%) | 283(72.2%) |  |
| Validation set | 25(8.3%) | 1444(9.8%) | 4792(9.7%) | 121(8.9%) | 25(6.4%) |  |
| Test set | 69(22.9%) | 2927(19.8%) | 9850(19.9%) | 292(21.4%) | 84(21.4%) |  |
| **Heart rate** | 88.5±20.0 | 86.0±21.3 | 83.9±21.6 | 88.4±25.9 | 85.3±27.1 | <0.0001 |
| **PR interval** | 165.5±46.5 | 163.0±34.4 | 163.7±34.8 | 168.3±41.9 | 174.2±48.4 | <0.0001 |
| **QRS duration** | 103.6±20.7 | 100.4±21.0 | 99.4±20.8 | 105.1±24.9 | 117.7±31.6 | <0.0001 |
| **QT interval** | 425.9±81.1 | 394.3±54.9 | 388.8±50.2 | 389.0±61.7 | 407.4±74.6 | <0.0001 |
| **Corrected QT interval** | 505.5±68.9 | 462.2±46.4 | 449.6±40.7 | 456.8±42.5 | 466.8±55.3 | <0.0001 |
| **P wave axes** | 46.8±51.4 | 48.7±35.9 | 47.9±35.6 | 46.5±45.4 | 44.3±52.8 | 0.0369 |
| **RS wave axes** | 25.8±52.2 | 34.7±47.5 | 35.0±48.2 | 30.7±58.8 | 33.6±73.9 | 0.0002 |
| **T wave axes** | 48.1±81.3 | 42.4±58.2 | 44.7±50.0 | 63.2±61.2 | 61.7±59.5 | <0.0001 |

The results of significant post hoc analysis (Bonferroni method) are as follows:

K^+^ ≤ 2.5 vs. 3.5 < K^+^ < 5.5: Heart rate, QRS duration, QT interval, Corrected QT interval, and RS wave axes; 2.5 < K^+^ ≤ 3.5 vs. 3.5 <K^+^ < 5.5: Heart rate, QRS duration, QT interval, Corrected QT interval, and T wave axes; 5.5 ≤ K^+^ < 6.5 vs. 3.5 <K^+^ < 5.5: Heart rate, PR interval, QRS duration, Corrected QT interval, RS waves axes, and T wave axes; 6.5 ≤ K^+^ vs. 3.5 < K^+^ < 5.5: PR interval, QRS duration, QT interval, Corrected QT interval, and T wave axes.

**Supplementary Table MA1-3. Model performance for each lead in the test set (n = 13,222).**

| **Value (95% CI)** | **MAE** | **hypokalemia** | | | | **hyperkalemia** | | | | **Kappa** | **Kappa** |
| --- | --- | --- | --- | --- | --- | --- | --- | --- | --- | --- | --- |
|  |  | **sens** | **spec** | **ppv** | **npv** | **sens** | **spec** | **ppv** | **npv** | **(3-class)** | **(5-class)** |
| **ECG lead** |  |  |  |  |  |  |  |  |  |  |  |
| Lead I | 0.843  (0.831-0.854) | 0.491  (0.473-0.509) | 0.685  (0.676-0.695) | 0.313  (0.300-0.326) | 0.821  (0.813-0.829) | 0.452  (0.402-0.505) | 0.904  (0.898-0.909) | 0.121  (0.105-0.138) | 0.983  (0.980-0.985) | 0.179  (0.162-0.194) | 0.187  (0.170-0.204) |
| Lead II | 0.784  (0.773-0.796) | 0.528  (0.512-0.546) | 0.693  (0.683-0.702) | 0.335  (0.321-0.349) | 0.834  (0.826-0.842) | 0.465  (0.413-0.514) | 0.913  (0.908-0.918) | 0.135  (0.117-0.156) | 0.983  (0.981-0.985) | 0.211  (0.195-0.228) | 0.227  (0.209-0.244) |
| Lead III | 0.981  (0.970-0.994) | 0.495  (0.478-0.513) | 0.693  (0.684-0.702) | 0.321  (0.308-0.333) | 0.824  (0.816-0.832) | 0.463  (0.413-0.511) | 0.837  (0.830-0.844) | 0.077  (0.066-0.088) | 0.982  (0.979-0.984) | 0.180  (0.166-0.195) | 0.185  (0.170-0.200) |
| aVR | 0.800  (0.789-0.812) | 0.382  (0.364-0.398) | 0.789  (0.781-0.797) | 0.346  (0.329-0.361) | 0.813  (0.805-0.821) | 0.532  (0.485-0.580) | 0.872  (0.866-0.878) | 0.109  (0.095-0.123) | 0.985  (0.982-0.987) | 0.191  (0.175-0.208) | 0.216  (0.199-0.234) |
| aVL | 0.902  (0.890-0.913) | 0.556  (0.538-0.573) | 0.568  (0.559-0.577) | 0.274  (0.264-0.284) | 0.814  (0.805-0.823) | 0.356  (0.308-0.404) | 0.914  (0.910-0.919) | 0.108  (0.092-0.126) | 0.980  (0.977-0.982) | 0.127  (0.111-0.143) | 0.138  (0.122-0.154) |
| aVF | 0.865  (0.853-0.876) | 0.487  (0.469-0.505) | 0.723  (0.713-0.731) | 0.340  (0.326-0.354) | 0.828  (0.819-0.835) | 0.487  (0.438-0.537) | 0.869  (0.864-0.876) | 0.098  (0.084-0.113) | 0.983  (0.981-0.985) | 0.200  (0.184-0.216) | 0.215  (0.198-0.232) |
| V1 | 0.843  (0.832-0.854) | 0.594  (0.578-0.612) | 0.620  (0.611-0.630) | 0.315  (0.303-0.326) | 0.839  (0.831-0.848) | 0.362  (0.315-0.409) | 0.918  (0.913-0.923) | 0.115  (0.096-0.132) | 0.980  (0.978-0.983) | 0.193  (0.178-0.207) | 0.200  (0.184-0.214) |
| V2 | 0.848  (0.837-0.860) | 0.468  (0.449-0.486) | 0.763  (0.755-0.771) | 0.366  (0.351-0.381) | 0.830  (0.822-0.837) | 0.527  (0.475-0.577) | 0.870  (0.864-0.876) | 0.106  (0.092-0.120) | 0.984  (0.982-0.987) | 0.235  (0.218-0.251) | 0.249  (0.233-0.265) |
| V3 | 0.775  (0.764-0.786) | 0.452  (0.434-0.470) | 0.769  (0.761-0.776) | 0.364  (0.349-0.377) | 0.827  (0.819-0.834) | 0.503  (0.447-0.556) | 0.908  (0.903-0.913) | 0.137  (0.118-0.156) | 0.984  (0.982-0.986) | 0.239  (0.223-0.256) | 0.259  (0.242-0.276) |
| V4 | 0.833  (0.822-0.844) | 0.494  (0.475-0.511) | 0.735  (0.725-0.742) | 0.353  (0.338-0.367) | 0.832  (0.823-0.840) | 0.513  (0.464-0.567) | 0.881  (0.876-0.887) | 0.112  (0.098-0.128) | 0.984  (0.982-0.986) | 0.230  (0.214-0.247) | 0.253  (0.236-0.269) |
| V5 | 0.742  (0.732-0.752) | 0.459  (0.441-0.476) | 0.746  (0.737-0.754) | 0.346  (0.331-0.361) | 0.825  (0.817-0.833) | 0.492  (0.443-0.542) | 0.916  (0.911-0.920) | 0.146  (0.126-0.164) | 0.984  (0.982-0.986) | 0.219  (0.203-0.236) | 0.248  (0.229-0.266) |
| V6 | 0.748  (0.736-0.759) | 0.405  (0.387-0.422) | 0.802  (0.795-0.810) | 0.375  (0.359-0.392) | 0.821  (0.814-0.829) | 0.516  (0.470-0.565) | 0.913  (0.908-0.918) | 0.148  (0.129-0.167) | 0.985  (0.982-0.987) | 0.235  (0.217-0.252) | 0.261  (0.242-0.279) |
| **ECG12Net-1** | 0.531  (0.523-0.539) | 0.507  (0.488-0.524) | 0.816  (0.808-0.823) | 0.447  (0.430-0.462) | 0.850  (0.842-0.856) | 0.508  (0.458-0.557) | 0.960  (0.956-0.963) | 0.269  (0.235-0.303) | 0.985  (0.983-0.987) | 0.354  (0.338-0.370) | 0.396  (0.379-0.415) |
| **EMPNet (SVM)** | 0.699  (0.690-0.710) | 0.237  (0.222-0.253) | 0.858  (0.851-0.865) | 0.316  (0.297-0.336) | 0.802  (0.794-0.809) | 0.264  (0.221-0.309) | 0.936  (0.932-0.940) | 0.098  (0.079-0.117) | 0.979  (0.976-0.981) | 0.117  (0.105-0.130) | 0.130  (0.116-0.146) |
| **EMPNet (RF)** | 0.565  (0.557-0.573) | 0.034  (0.028-0.041) | 0.987  (0.985-0.989) | 0.429  (0.366-0.494) | 0.787  (0.780-0.794) | 0.004  (0.000-0.013) | 0.999  (0.998-1.000) | 0.143  (0.000-0.357) | 0.974  (0.971-0.977) | 0.032  (0.025-0.041) | 0.033  (0.025-0.042) |
| **EMPNet (XGB)** | 0.603  (0.592-0.613) | 0.254  (0.244-0.264) | 0.788  (0.780-0.796) | 0.249  (0.240-0.258) | 0.783  (0.775-0.790) | 0.040  (0.036-0.044) | 0.991  (0.988-0.994) | 0.119  (0.104-0.124) | 0.972  (0.970-0.974) | 0.049  (0.039-0.062) | 0.054  (0.044-0.067) |
| **EMPNet (MLP)** | 0.605  (0.595-0.618) | 0.131  (0.126-0.136) | 0.930  (0.926-0.934) | 0.341  (0.327-0.355) | 0.785  (0.777-0.792) | 0.088  (0.083-0.093) | 0.986  (0.983-0.990) | 0.160  (0.141-0.179) | 0.974  (0.972-0.976) | 0.096  (0.083-0.111) | 0.100  (0.087-0.115) |
| **ECG12Net-2** | 0.538  (0.531-0.547) | 0.511  (0.492-0.529) | 0.813  (0.806-0.821) | 0.444  (0.429-0.461) | 0.848  (0.840-0.855) | 0.511  (0.462-0.558) | 0.959  (0.956-0.962) | 0.267  (0.234-0.297) | 0.985  (0.983-0.987) | 0.345  (0.329-0.361) | 0.392  (0.373-0.410) |

MAE: mean absolute error. sens, spec, ppv, and npv: sensitivity (recall), specificity, positive predictive value (precision), and negative predictive value, respectively, of hypokalemia (K^+^ ≤ 3.5) or hyperkalemia (5.5 ≤ K^+^). These values were calculated from binary variables converted from linear model predictions. Kappa: squared weighted kappa calculated from 3 classifications (K^+^ ≤ 3.5, 3.5 < K^+^ < 5.5, 5.5 ≤ K^+^) and 5 classifications (K^+^ ≤ 2.5, 2.5 < K^+^ ≤ 3.5, 3.5 < K^+^ < 5.5, 5.5 ≤ K^+^ < 6.5, and 6.5 ≤ K^+^). EMPNet (SVM): support vector machine with radial basis kernel function trained by EMPs and K^+^ concentration; EMPNet (RF): random forest trained by EMPs and K^+^ concentration; EMPNet (XGB): eXtreme gradient boosting model trained by EMPs and K^+^ concentration; EMPNet (MLP): multilayer perceptron trained by EMPs and K^+^ concentration. The ECG12Net-1 made predictions based on only ECG data, and the ECG12Net-2 made predictions based on ECG data and the EMPs from EMPNet.


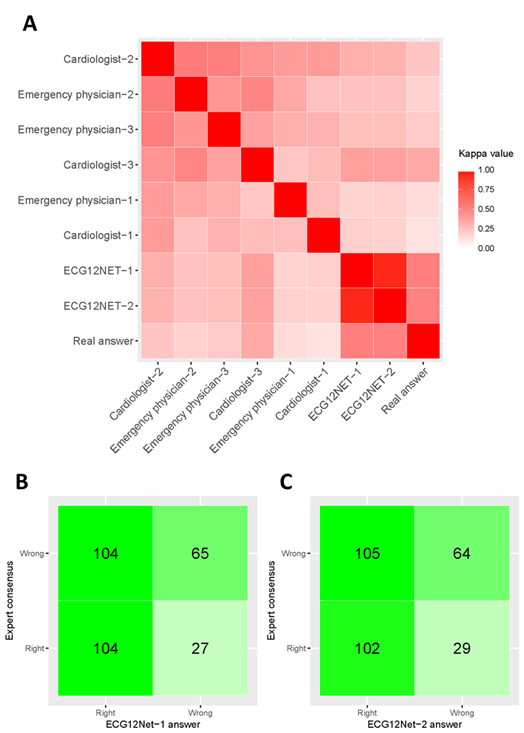


**Supplementary Figure 1. Consistency analysis of answers given by ECG12Net and human experts.** Supplementary Figure 1-A shows a consistency heatmap with kappa values. The answers given by the experts were highly consistent, but the consistency between the experts’ answers and the real answers was low. Compared with those of the human experts, the answers given by ECG12Net showed greater consistency with the real answers. Supplementary Figure 1-B and Supplementary Figure 1-C show the results of pair analysis of the expert consensus and the output of ECG12Net. ECG12Net was more accurate than the expert consensus. In addition, the expert consensus and ECG12Net showed approximately 45% divergence, and the answers yielded by ECG12Net were more likely to be correct.


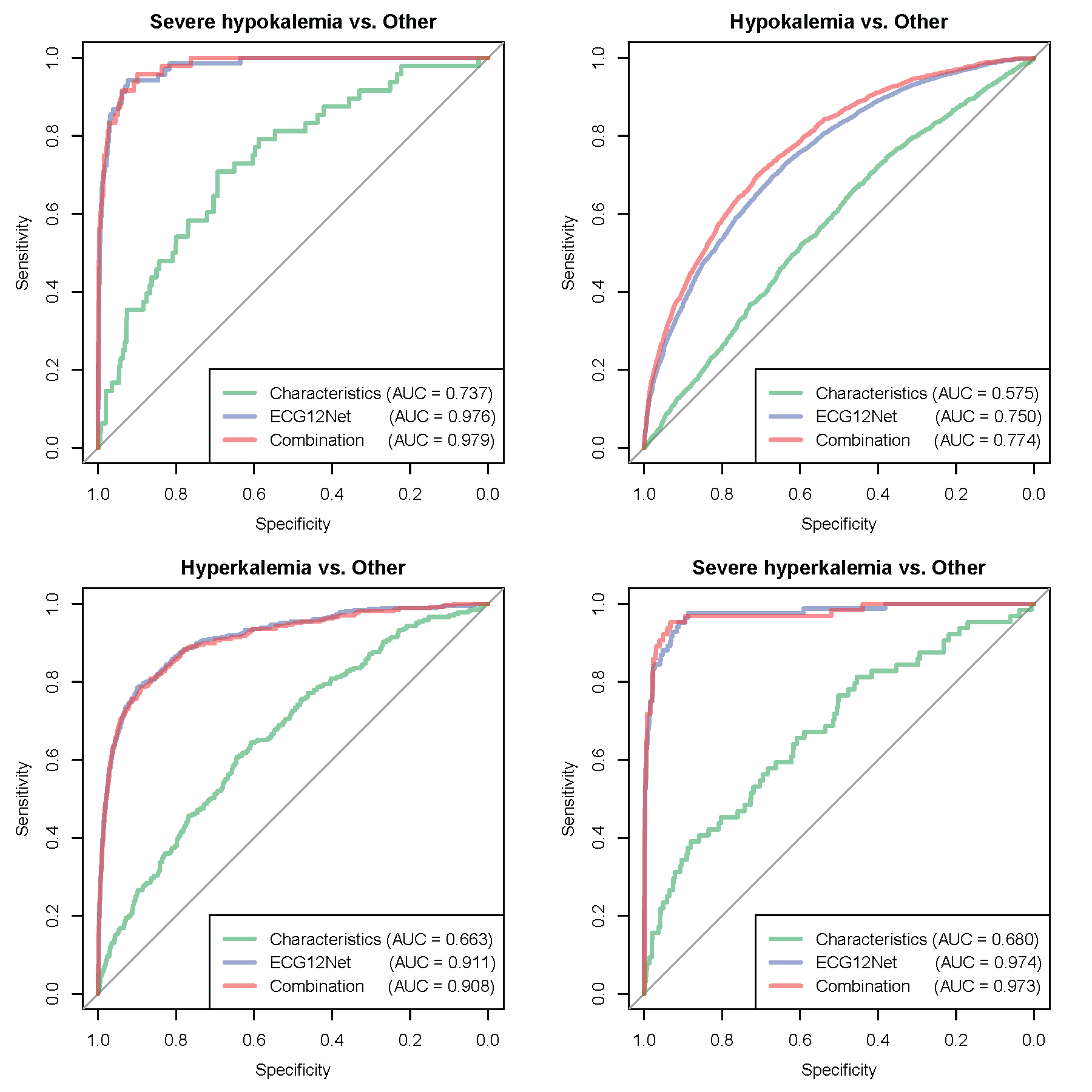


**Supplementary Figure 2. Performance in detecting dyskalemia in the test set (n = 13,222), ECG12Net versus patient characteristics.** These ROC curves were calculated from the logistic regression models. The first model uses the patient demographic characteristics only, including gender, age, BMI, and comorbidities (DM, CAD, hypertension, heart failure, hyperlipidemia, CKD, COPD, and pneumothorax). The second model uses the ECG12Net-1 prediction value. The third model uses the combination of the patient characteristics and the ECG12-1 prediction value.

**Clinical applications of ECG12Net on dyskalemia ECG detection**

**Case 1** A 66-year-old man with diabetic nephropathy in the uremic state on regular hemodialysis for 4 years presented to the emergency department (ED) with near fainting in the morning. He underwent AV shunt thrombectomy due to malfunction of the AV shunt one week prior to this visit. On physical examination, hypotension (80/50 mmHg) was noted. The ECG revealed a junctional rhythm with a rate of 35 beats per min and tall T waves in V4-V6 (Supplementary Fig 3, upper panel). A presumptive diagnosis of acute coronary artery syndrome or hyperkalemia was made clinically. The ECG was interpreted by ECG12Net (Supplementary Fig. 3, lower panel), which demonstrated an extremely high K^+^ concentration (6.9 to 7.2). Potassium-lowering agents along with emergent hemodialysis were initiated. Laboratory results revealed hyperkalemia with serum K^+^ up to 7.5 mEq/L. The ECG returned a normal sinus rhythm after one hour of hemodialysis. The hyperkalemia of this patient was due to inadequate hemodialysis related to a malfunction of the AV shunt.


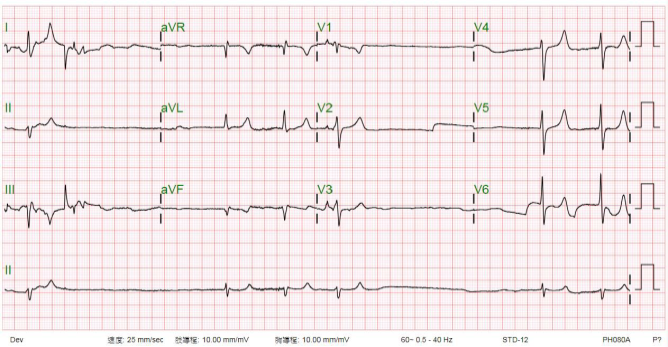


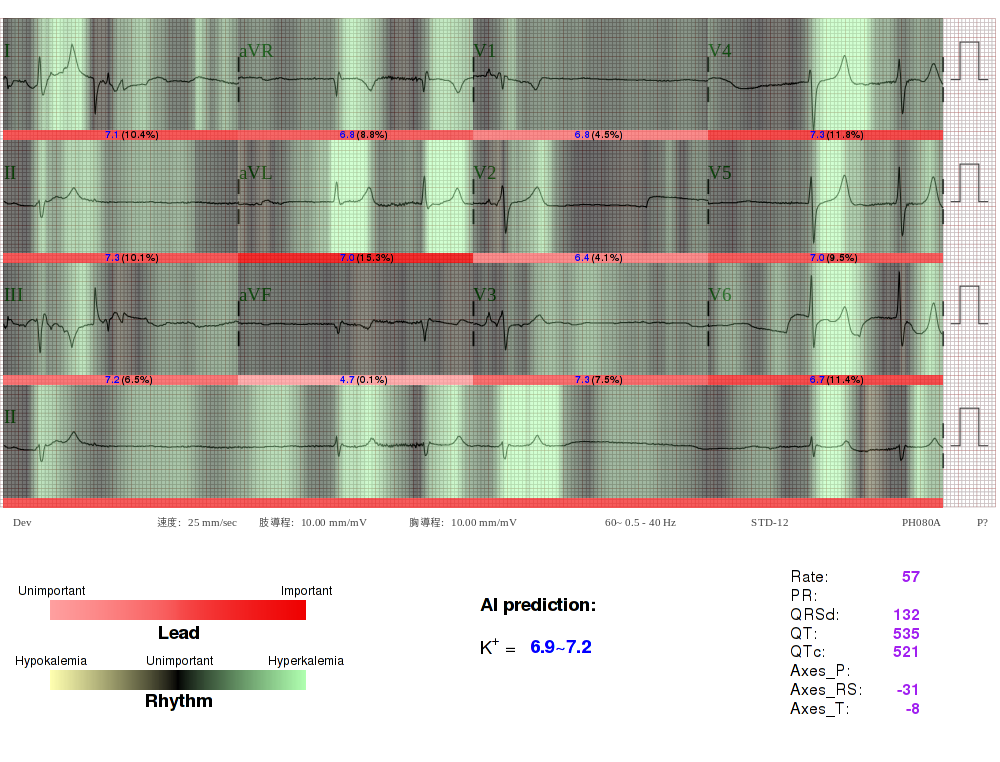


**Supplementary Figure 3.** ECGs in a patient with hyperkalemia presenting with near fainting

**Case 2** A 25-year-old lady presented to the ED with weakness to paralysis of the lower limbs upon waking in the morning. On physical examination, her blood pressure was 120/80 mmHg. She was alert an, clear with a lower limb muscle power of 3. The ECG revealed a normal sinus rhythm with nonspecific ST-T wave changes and a prolonged corrected QT interval (Supplementary Fig. 4, upper panel). A very low K^+^ concentration (1.7 to 1.9) was predicted by ECG12Net (Supplementary Fig. 4, lower panel). Aggressive K^+^ supplementation at 20 mEq/hour was initiated. Laboratory results revealed severe hypokalemia (K^+^=1.7 mEq/L) and normal anion gap metabolic acidosis (pH=7.314). The patient’s muscle power returned to normal after 200 mEq potassium supplement. She was discharged with a diagnosis of Sjögren syndrome with distal renal tubular acidosis complicated with hypokalemia paralysis.


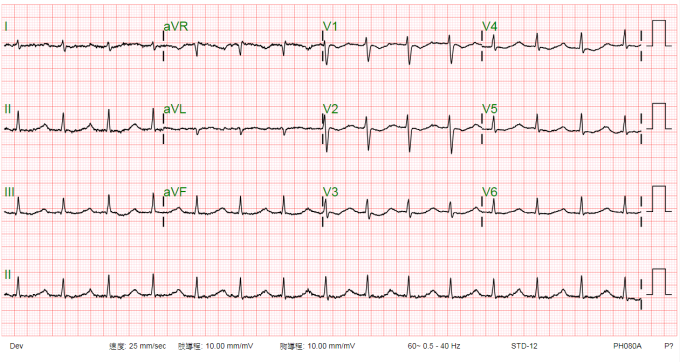


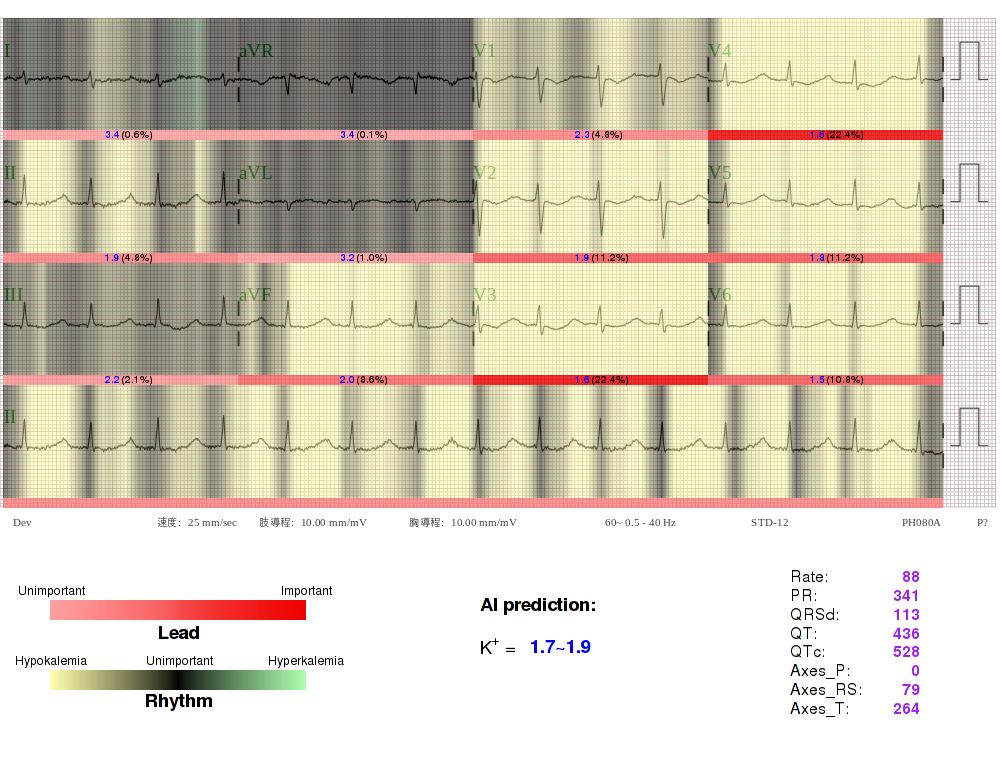


**Supplementary Figure 4.** ECGs in a patient with hypokalemia paralysis

**Case 3** A 47-year-old man presented to the ED with dyspnea and four limb edema for one day. On physical examination, his blood pressure was 116/86 mmHg. Pale conjunctiva and grade II pitting edema were found. Emergent laboratory results indicated marked hyperkalemia (9.1 mEq/L) without hemolysis. Emergent hemodialysis was planned. However, the ECG demonstrated a normal sinus rhythm without significant ST-T wave changes (Supplementary Fig. 5, upper panel). A normal serum K^+^ concentration was predicted by ECG12Net (Supplementary Fig. 5, lower panel), suggesting the presence of pseudohyperkalemia. Meanwhile, other laboratory data revealed leukocytosis (WBC 541.4x10^3^/μL, normal range 4-11x10^3^/μL), anemia (hemoglobin 4.9 g/dL, normal range 13-17 g/dL), thrombocytopenia (platelet 14x10^3^/μL, normal range 150-400x10^3^/μL) and normal renal function (creatinine 0.9 mg/dL). The patient was diagnosed with acute T cell lymphoblastic leukemia. His pseudohyperkalemia was due to fragile leukemia cells with lysis of white blood cells during blood collection and vacuum tube transportation.


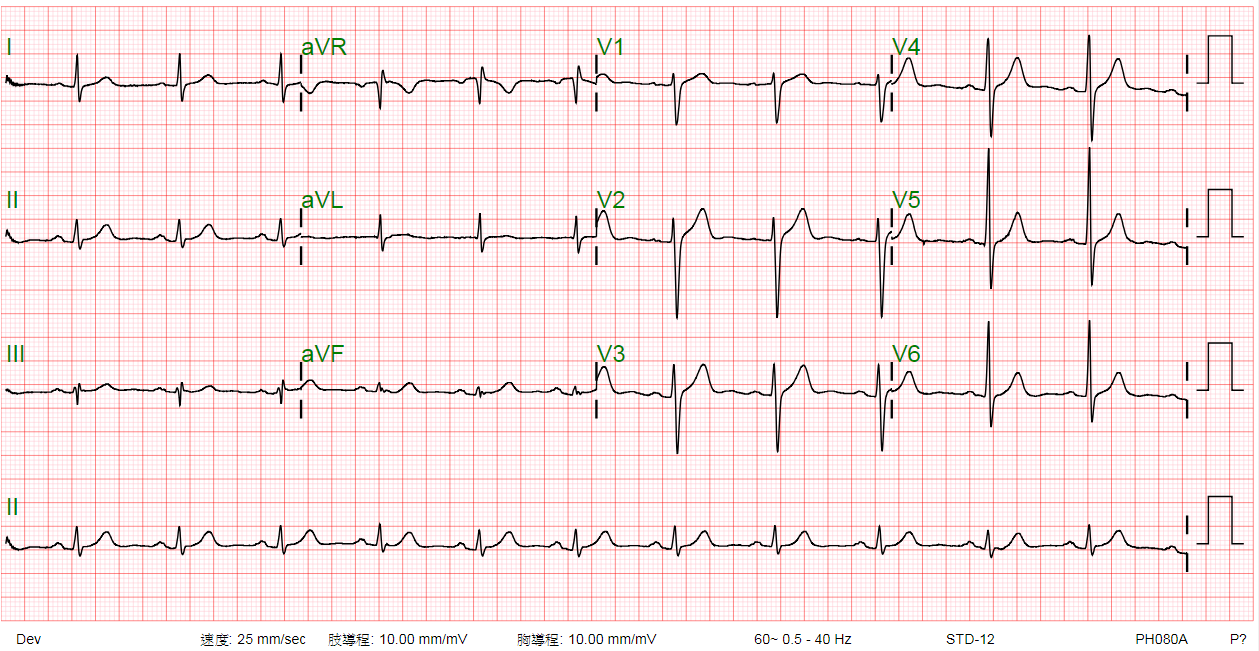


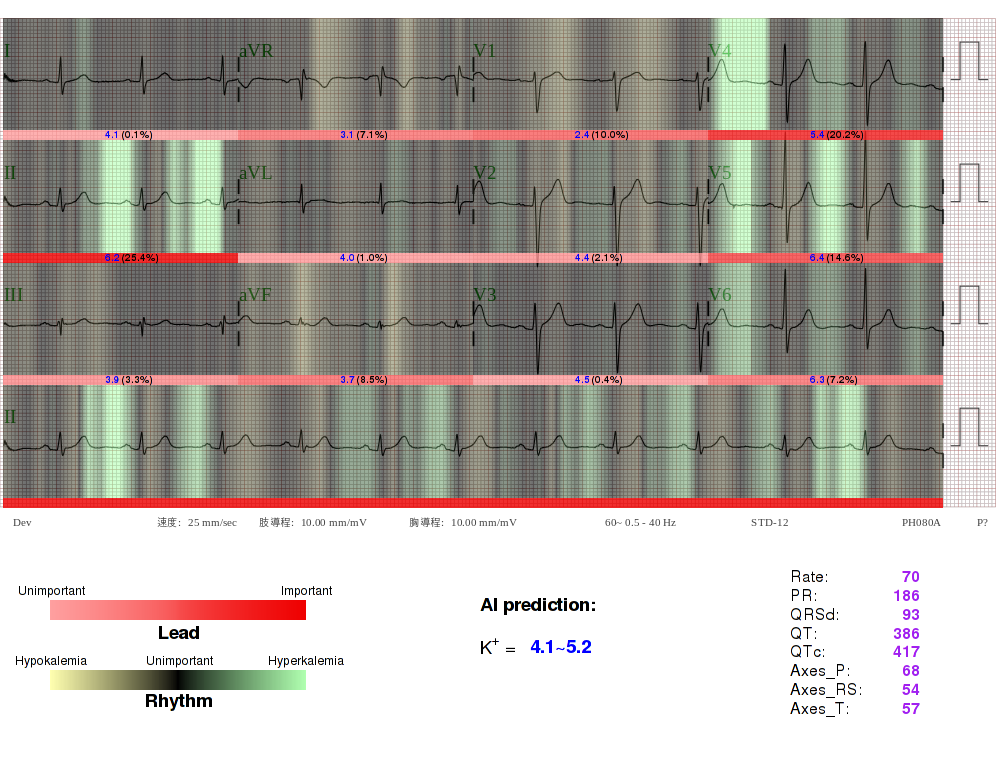


**Supplementary Figure 5.** ECGs in a patient with pseudohyperkalemia

**References**

1. Huang G, Liu Z, Weinberger KQ, van der Maaten L. Densely connected convolutional networks. In: *The IEEE Conference on Computer Vision and Pattern Recognition*. *2017*.

2. Ioffe S, Szegedy C. Batch normalization: Accelerating deep network training by reducing internal covariate shift. In: *International Conference on Machine Learning*. *2015*, p. 448-456.

3. Glorot X, Bordes A, Bengio Y. Deep sparse rectifier neural networks. In: *Proceedings of the Fourteenth International Conference on Artificial Intelligence and Statistics*. *2011*, p. 315-323.

4. LeCun Y, Bottou L, Bengio Y, Haffner P. Gradient-based learning applied to document recognition. Proceedings of the IEEE 1998;**86**(11):2278-2324.

5. Yang Z, Yang D, Dyer C, He X, Smola AJ, Hovy EH. Hierarchical Attention Networks for Document Classification. In: *HLT-NAACL*. *2016*, p. 1480-1489.

6. Srivastava N, Hinton GE, Krizhevsky A, Sutskever I, Salakhutdinov R. Dropout: a simple way to prevent neural networks from overfitting. Journal of machine learning research 2014;**15**(1):1929-1958.

7. Yang H-F, Lin B-Y, Chang K-Y, Chen C-S. Automatic age estimation from face images via deep ranking. networks 2013;**35**(8):1872-1886.

8. Lin C, Hsu CJ, Lou YS, Yeh SJ, Lee CC, Su SL, Chen HC. Artificial Intelligence Learning Semantics via External Resources for Classifying Diagnosis Codes in Discharge Notes. J Med Internet Res 2017;**19**(11):e380.

9. Simpson AJ. Over-sampling in a deep neural network. arXiv preprint arXiv:1502.03648 2015.

10. Chen T, Li M, Li Y, Lin M, Wang N, Wang M, Xiao T, Xu B, Zhang C, Zhang Z. Mxnet: A flexible and efficient machine learning library for heterogeneous distributed systems. arXiv preprint arXiv:1512.01274 2015.

11. Gross S, Wilber M. Training and investigating residual nets. Facebook AI Research, CA.[Online]. Avilable: <http://torch>. ch/blog/2016/02/04/resnets. html 2016.

12. Zihlmann M, Perekrestenko D, Tschannen M. Convolutional Recurrent Neural Networks for Electrocardiogram Classification. arXiv preprint arXiv:1710.06122 2017.

13. Zhou B, Khosla A, Lapedriza A, Oliva A, Torralba A. Learning deep features for discriminative localization. In: *Proceedings of the IEEE Conference on Computer Vision and Pattern Recognition*. *2016*, p. 2921-2929.

14. Mansi IA, Nash IS. Ethnic differences in electrocardiographic intervals and axes. J Electrocardiol 2001;**34**(4):303-7.
